# Use of complementary and alternative medicine in Germany – a survey of patients with inflammatory bowel disease

**DOI:** 10.1186/1472-6882-6-19

**Published:** 2006-05-22

**Authors:** Stefanie Joos, Thomas Rosemann, Joachim Szecsenyi, Eckhart G Hahn, Stefan N Willich, Benno Brinkhaus

**Affiliations:** 1 Department of General Practice and Health Services Research, University of Heidelberg, Germany; 2 Department of Medicine I, Friedrich Alexander University of Erlangen Nuremberg, Erlangen, Germany; 3 Institute of Social Medicine, Epidemiology, and Health Economics, Charité University Medical Center, Berlin, Germany

## Abstract

**Background:**

Previous studies have suggested an increasing use of complementary and alternative medicine (CAM) in patients with inflammatory bowel disease (IBD). The aim of our study was to evaluate the use of CAM in German patients with IBD.

**Methods:**

A questionnaire was offered to IBD patients participating in patient workshops which were organized by a self-help association, the German Crohn's and Colitis Association. The self-administered questionnaire included demographic and disease-related data as well as items analysing the extent of CAM use and satisfaction with CAM treatment. Seven commonly used CAM methods were predetermined on the questionnaire.

**Results:**

413 questionnaires were completed and included in the analysis (n = 153 male, n = 260 female; n = 246 Crohn's disease, n = 164 ulcerative colitis). 52 % of the patients reported CAM use in the present or past. In detail, homeopathy (55%), probiotics (43%), classical naturopathy (38%), Boswellia serrata extracts (36%) and acupuncture/Traditional Chinese Medicine (TCM) (33%) were the most frequently used CAM methods. Patients using probiotics, acupuncture and Boswellia serrata extracts (incense) reported more positive therapeutic effects than others. Within the statistical analysis no significant predictors for CAM use were found. 77% of the patients felt insufficiently informed about CAM.

**Conclusion:**

The use of CAM in IBD patients is very common in Germany, although a large proportion of patients felt that information about CAM is not sufficient. However, to provide an evidence-based approach more research in this field is desperately needed. Therefore, physicians should increasingly inform IBD patients about benefits and limitations of CAM treatment.

## Background

The use of complementary and alternative medicine (CAM) is widespread and still increasing in the Western world [1,2]. The term complementary medicine refers to a group of diagnostic and therapeutic disciplines that exist largely outside the institutions where conventional health care is provided and taught [3]. The wide range of disciplines classified as complementary medicine makes it difficult to find defining criteria that are common to all. Furthermore, the diagnostic and therapeutic approaches which are summarized among the term CAM depend on traditions and historical developments of a country and therefore, vary considerably among countries [4]. Also, the extent to which CAM is practiced by physicians or non-medical therapists differs considerably among countries. In Germany, medical doctors can obtain a variety of additional qualifications relating to specific CAM methods (e.g. chiropractic, homeopathy) or to naturopathic medicine in general (naturopathy). In 2004 the German federal medical chamber documented 15.970 additional qualifications of medical doctors in chiropractic, 5.538 in homeopathy and 13.502 in naturopathy (= 18.5% physicians working in the ambulatory sector). In addition, about 2.5% of the ambulatory physicians are qualified in the field of physical medicine [5]. Furthermore, around 25% of the physicians in the ambulatory sector have passed a special training in acupuncture. In near future, acupuncture will also be a qualification accredited by the local medical chambers such as chiropractic or homeopathy. Moreover, in Germany many physicians are providing CAM in their daily practice without having an additional CAM qualification [6].

Other health care professionals such as pharmacists, dentists, physiotherapists or midwives also practise some form of CAM. Official data on CAM qualifications for these health care professions are not available.

Apart from the regular health care professionals Germany has one state-regulated profession – the 'Heilpraktiker'. A 'Heilpraktiker' has to pass an examination on basic medical knowledge and skills at a local public health office to obtain a state license. 'Heilpraktiker' only practice in the ambulatory sector and their services are not covered by health insurance funds. Any CAM method can be performed by a 'Heilpraktiker' as long as it is consistent with the general standards of good professional practice in health care as supervised by the local public health office. In general 'Heilpraktiker' provide a variety of CAM methods. Within the last ten years the number of 'Heilpraktiker' increased from 9.000 in the year 1993 to nearly 20.000 today.

In Germany, the overall percentage of individuals with CAM experience increased from 52% in 1970 to 73% in 2002 [5]. A recently published national representative sample shows that herbal medicine, exercise therapy and hydrotherapy are the most frequently used CAM methods [7] – all of them belonging to the so-called classic naturopathy, also known as kneippism from its originator Sebastian Kneipp (1821–97), a German priest. Besides the classic naturopathic methods homeopathy, manual therapy and acupuncture are commonly used CAM therapies in Germany [7].

It is commonly known, that patients with chronic diseases rank high among CAM users. Several surveys in patients with IBD have shown rates of CAM utilization ranging from 39% in Austria to 47% in Switzerland and Canada [8-11]. A survey among German IBD patients found 51% with experience in CAM. Homeopathy and herbal medicine were the most commonly used types of CAM in this recently published survey [12].

The purpose of our study was to estimate the extent of CAM experience among IBD patients in Germany and to obtain information about the most commonly used CAM methods, about CAM providers and about perceived effects of CAM therapy.

## Methods

### Participants and data collection

We surveyed IBD patients meeting at patient workshops organized by the German Crohn's and Ulcerative Colitis Association (*DCCV e.V*.). The DCCV [13] is a national organization established by patients and for patients with Crohn's disease (CD) and Ulcerative Colitis (UC). The patient workshops of the DCCV take place regularly in different regions of Germany and are open to the public. Normally experts are invited to give lectures on recent developments in diagnostic and therapeutic possibilities in order to inform patients. Three of these workshops (which were not specific to CAM) took place in different German federal states (Erlangen; October 1998/Aachen; May 2000/Tübingen; June 2000). A questionnaire was handed out to all participants of the workshops. The questionnaires were distributed on the tables in the convention hall and had to be completed during the workshop or were to be returned by mail.

### Questionnaire

The 4-page structured self-administered questionnaire contained questions on patients' demographic data (10 items) and on characteristics of the disease such as duration of disease, current medication and satisfaction with conventional therapy (16 items). Detailed questions were asked about knowledge and interest in CAM and CAM use specifically for their IBD symptoms (15 items). The items were designed as yes/no questions or open-ended questions or to be answered on a 5-point Likert scale.

Within the questionnaire CAM was defined as 'all non-conventional therapy methods, which are based rather on personal experience than on a scientific basis'. Seven CAM therapies which are commonly used in Germany were predetermined in the questionnaire: classic naturopathic medicine (incl. herbal medicine and hydrotherapy), Traditional Chinese Medicine incl. acupuncture (TCM), homeopathy, Boswellia serrata (incense), probiotics, neural therapy (= pain therapy with local anaesthetics) and anthroposophic medicine.

Patients were asked to assess the perceived therapeutic effect of CAM treatment on a 5-point Likert scale and to indicate where they received the CAM therapy: general practitioner, gastroenterologist, clinician or 'Heilpraktiker" (= state registered non-physician CAM practitioner).

### Data analysis

Data analysis was completed with SPSS (Version 9.0). Descriptive statistics (mean and proportions) were used to describe each variable. The chi-square test was used to identify significant differences in CAM use regarding possible predictive patient characteristics such as age, gender, weight, graduation level, membership of DCCV, site of workshop, disease, current medication, smoking status, special diets, regular exercise or type of health insurance. Since no significant differences were found for any of these variables during statistical analysis, data in the result section will be presented for the complete sample only.

Due to the fact that probiotic therapy was missing on the list of predetermined CAM methods within the Erlangen questionnaire, the percent values for probiotic use were calculated for the sample without Erlangen patients.

## Results

### Respondents

According to information given by the German Crohn's and Ulcerative Colitis Association about 250 patients participated at the workshop in Aachen, 250 patients in Tübingen and about 340 patients in Erlangen (n _total _= 840). Altogether 413 patients completed and returned the questionnaire which corresponds to an overall response rate of 49% (Erlangen 61%, Aachen 35%, and Tübingen 47%). All 413 questionnaires were included in the final analysis sample.

All in all there was a greater proportion of patients with Crohn's disease (n = 246) than with Ulcerative Colitis (n = 164) returning the questionnaire. 3 patients did not indicate their diagnosis. Table 1 shows the demographic, disease-related and treatment characteristics. Duration of disease was 14 ys in average and patients consulted their general practitioner about 12 times a year for their IBD. About 80% of the patients had been hospitalised because of IBD at least once and 39% had undergone some kind of surgical intervention for their IBD. 45% of the patients stated that they were satisfied with their current conventional treatment: 64% of the patients receiving aminosalycilates, 36% being treated with corticosteroids and 17% taking immunosuppressive drugs (Table 1). 55% of the IBD patients reported side-effects due to their conventional treatment. 25% of the patients were on a special diet (vegetable diet, sugar-reduced diet, whole foods etc.). On a 5 -point Likert scale 32 % of the patients indicated that they feel stressed or highly stressed by their disease on a physical level and 36% on a mental level.

**Table 1:** Sociodemographic and disease-related characteristics of the study population.

***Characteristics***	***All Patients***	***CAM user ***	***CAM non-user ***	***P value***^**#**^
	*****(n = 413)*	*(n = 215)*	*(n = 198)*	
Sex (%)				
Male	38	39	37	ns
Female	62	61	63	ns
Age (%)				
≤20	6	5	8	ns
21–40	56	56	56	ns
41–60	32	33	30	ns
≥61	6	6	5	ns
Diagnosis (%)				
Crohn's Disease	61	58	65	ns
Ulcerative Colitis	39	42	35	ns
Location (% of location)				
Erlangen	50	55	45	ns
Aachen	21	53	47	ns
Tuebingen	28	49	51	ns
Duration of disease (median)	14.0	15	13	ns
Previous hospitalisation (%)	80	82	77	ns
Previous surgery (%)	39	36	41	ns
Current Medication (%)				
SASP or 5-ASA	64	54	72	ns
Corticosteroids	36	32	40	ns
Immunosuppressive	17	17	16	ns
Patient satisfaction with current therapy (%)				
satisfied	45	43	48	ns
neutral	31	30	32	ns
dissatisfied	19	17	20	ns
Side effects of conventional medication (%)	55	58	52	ns
Members of DCCV (%)*	48	47	48	ns
Academic education (%)*	23	22	24	ns

If not stated otherwise, all values are percentages of CAM users and CAM non-users resp.

^# ^p value for the comparison of CAM users and non-users

*This item was missing for Erlangen patients.

For some items the numbers may not add up to the total number because of missing responses

Comparing the three sites Erlangen (n = 207), Aachen (n = 88) and Tübingen (n = 118) some small, however not significant differences were found. Tübingen-patients had been slightly less hospitalised and had undergone fewer surgical interventions compared to patients in Erlangen and Aachen. In addition, the application of pharmacotherapy, in particular aminosalicylates, was slightly lower among Tübingen-patients.

### Use and provider of CAM

52% (n = 215) of all patients reported the use of some form of CAM to treat their IBD currently or in the past (Table 2). The most frequently used therapy method was homeopathy (55%), followed by probiotics (43%), classical naturopathy (38%), Boswellia-serrata extracts (36%) and acupuncture/TCM (33%). Anthroposophic medicine (7%) and neural therapy (5%) were applied less frequently.

**Table 2:** Previous or present use of CAM

	**Patients using CAM**
**Classical naturopathy **n (% of CAM users)	82 (38 %)
**Boswellia serrata **n (% of CAM users)	78 (36 %)
**Probiotics **n (% of CAM users)	44 (43 %)*
**Acupuncture/TCM **n (% of CAM users)	70 (33 %)
**Homeopathy **n (% of CAM users)	119 (55 %)

* calculated for n = 102 CAM users, because this item was missing in the Erlangen questionnaire.

CAM was initiated in 40% by a general practitioner, in 29% by a practicing gastroenterologist and in 15% by a clinician. 44% of the patients visited a so-called 'Heilpraktiker" for the CAM treatment. 54% of those patients, who received CAM treatment not by their general practitioners, informed their GP about the additional treatment with CAM. There were no significant differences in CAM use regarding any patient-related characteristics.

#### Benefit, risks and costs of CAM

Within the questionnaire patients were asked on a 5-point Likert scale how satisfied they were regarding CAM treatment for IBD. Most satisfied were patients treated with probiotics (57%), followed by patients treated with acupuncture/TCM (49%) and Boswellia serrata extract (44%) (Table 3). 15% of the CAM users notified side effects of the CAM treatment, in general. However, the questionnaire did not assess side effects of specific CAM methods in detail.

**Table 3:** Subjective rating of the efficacy of CAM treatment

	**Satisfied with CAM treatment **	**No change **	**Unsatisfied with CAM treatment **
	*n (%)*	*n (%)*	*n (%)*
Classical naturopathy (n = 82)	28 (34%)	17 (21%)	30 (37%)
Boswellia serrata extract (n = 78)	34 (44%)	9 (12%)	21 (27%)
Probiotics (n = 44)	25 (57%)	6 (14%)	7 (16%)
Acup./TCM (n = 70)	34 (49%)	15 (21%)	14 (20%)
Homeopathy (n = 119)	47 (39%)	28 (24%)	42 (35%)

73% of the patients receiving CAM reported that they financed the treatment in part or completely themselves. Only 23% of all patients felt sufficiently informed about CAM treatment in IBD. Most respondents required that CAM should be reimbursed by the statutory health insurance (96%) and that research in CAM should be increased (97%).

## Discussion

52% of the 413 patients participating in our survey reported some form of current or previous CAM use for their IBD. These findings correspond to results in previous surveys in selected groups of IBD patients attending specialty clinics in North America and European countries [8,10,11,14]. In our survey homeopathy was the most frequently used CAM method, followed by probiotics, classical naturopathy (including herbal therapy), Boswellia serrata extracts and acupuncture/TCM. However, it is difficult to compare surveys of CAM utilization among countries because the extension of specific CAM methods is tightly linked to the traditional and cultural background of a country. While herbal products are very popular both in North America and in Europe, homeopathy is less used in North America, but very popular in Europe. As expected, the most commonly used CAM methods among our IBD patients are comparable with data from the neighbouring countries Austria and Switzerland [9,10]. In both of these countries as well as in the German survey of Langhorst et al. homeopathy was the most frequently used CAM method [12].

Various previous surveys have indicated that CAM users are generally more likely to be female and to be better educated [7,15]. In our study, duration of disease, gender, age and previous surgery/hospitalisation were not predictive for the use of CAM. Nor we could confirm the findings of Langhorst et al that steroid medication and academic education are strong predictors for CAM use [12]. Furthermore our results do not support the assumption that our patients use CAM because of dissatisfaction with conventional treatment or side effect profile of conventional medication. This lack of CAM-predictors is in accordance with the Swiss survey of Quattropani et al [10]. Accordingly, due to our data IBD patients using CAM seem not so different from non-users of CAM – at least regarding the evaluated predictors. Nevertheless, there may be differences between CAM users and non-users in terms of patients' perceptions of disease, expectations towards treatment or preferences for shared decision making. These factors would be interesting to assess in future surveys.

In some of the prior studies patients were asked to state the perceived effects of CAM treatment [10,11]. In the survey of Hilsden 67% of present CAM users and 35% of prior CAM users resp. reported beneficial effects regarding their IBD symptoms [11]. In the Quattropani survey 61% of the patients reported an improvement while using CAM [10]. However, no further differentiation regarding specific CAM methods was reported in these studies. In our survey the perceived beneficial effects varied between 57% for probiotic therapy and 34% for treatment with classical naturopathy incl. herbals. Although explanatory power of this data is limited it can be regarded as an indicator for patients' satisfaction with a specific CAM method.

A strength of the study is that CAM was defined clearly within the questionnaire and that CAM methods most commonly used among German patients were predetermined in the questionnaire. Furthermore, the survey was based on patients participating at IBD workshops organized by a self-help association but open for public. In contrast, most of the previous studies only included patients visiting special IBD clinics [8,11,14]. We therefore surveyed a less pre-selected collective. However, it was not a non-random sample and therefore a bias can not be excluded, because our population may still not be in conformity with the general IBD population, as 46% of the surveyed patients were members of the self-help association DCCV. Those patients may have different strategies to manage their disease. For example, they might take more responsibility for their health-care and the management of their treatment. The study may therefore overestimate the use of CAM among IBD patients.

A limit of our study is that the response rate only could be estimated retrospectively, since the questionnaires were not handed out personally to each patient. It may be possible that some patients did not recognize the questionnaires or that patients interested in CAM rather tended to complete the questionnaire than patients not interested in CAM leading to a possible selection bias. However, this argument may be invalidated by a comparison of our results with the study of Langhorst et al. who surveyed a randomly drawn sample of German IBD patients [12]. We found high corresponding results regarding CAM use (52% vs. 51%) and the high popularity of homeopathy (53% vs. 55%).

The most common CAM therapy used by patients in our survey was homeopathy. This finding might be influenced by the fact that homeopathy was established by S. Hahnemann, a German physician. Since homeopathic treatment is not reimbursed by the statutory health insurance, homeopathic therapies often are self initiated by the patients, provided by 'Heilpraktiker' or by homeopaths (physicians with the additional qualification 'homeopathy') in private practice. Unfortunately, in our survey we did not ask which CAM therapy was administered by which type of practitioner or which CAM therapy was self initiated by the patient resp. Perceived effects of the homeopathic treatment were less positive compared to the other CAM methods. Interestingly, in a Medline search no clinical study assessing the efficacy of homeopathic preparations in patients with IBD could be found.

Our data shows a frequent use of probiotics. This may be the consequence of several recently published clinical studies with probiotic preparations in the treatment of UC and CD [16-18]. According to the positive results of those studies the usage of probiotics for maintenance of remission is recommended in the updated guideline for Diagnosis and Therapy of Ulcerative Colitis by the German Society of Digestive and Metabolic Disease and the Competence Network of IBD [19]. Therefore, in Germany probiotic therapy can be prescribed for the treatment of IBD and will be paid for by the statutory health insurance. Despite implementation of probiotics in the national guideline, this therapy is originated from the field of CAM and it is therefore an excellent example for an evidence-based integration of CAM into conventional treatment.

Acupuncture/TCM was also frequently used by our IBD patients. Considering the popularity of acupuncture the high number of physicians with an additional training in acupuncture therapy in Germany is not surprising. Moreover, also many 'Heilpraktiker' provide acupuncture. Nevertheless, at present there are only a few observational studies on the effect of acupuncture in UC [20,21] and one single randomised, controlled study in MC patients [22]. MC patients showed a decrease of disease activity as well as an improvement in quality of life after acupuncture treatment in this study.

One third of our patients reported a therapeutic approach with Boswellia serrata extracts/incense. Preparations from the gum resin of Boswellia serrata have been used as a traditional remedy in Ayurvedic medicine in India for the treatment of inflammatory diseases. In clinical trials promising results were observed in patients with rheumatoid arthritis, inflammatory bowel disease and bronchial asthma, but to date results from larger randomised controlled studies are missing [23-25]. The therapeutic approaches with Boswellia serrata may have been self initiated by the patients or recommended by 'Heilpratiker'.

55% of the patients reported side effects on their conventional medication and 20% were completely dissatisfied with their current conventional treatment. According to previous studies this might be the main reason for IBD patients to enter CAM treatment [8]. Side effects of CAM therapy were described by 14% of our IBD patients. Unfortunately, the questionnaire provided no further information about type or severity of the perceived CAM side effects. Only 23% of the IBD patients in our survey felt sufficiently informed about CAM.

## Conclusion

Physicians should address their patients straightforward regarding CAM and offer evidence-based information about specific CAM methods. On the basis of available scientific data they should aim at guiding patients through the 'CAM jungle'. At the same time, further clinical studies assessing the most commonly used CAM therapies are urgently needed. Research in CAM offers the chance to discover new treatment options in the management of IBD but may also protect patients from ineffective and expensive 'pseudo'-therapies.

## Competing interests

The author(s) declare that they have no competing interests.

## Authors' contributions

SJ, BB, TR, JS and SW conceived the study and draft the manuscript. SJ, BB and EH participated in designing the study. All authors read and approved the final version of the manuscript.

## Pre-publication history

The pre-publication history for this paper can be accessed here:


